# Drivers of change in China’s energy-related CO_2_ emissions

**DOI:** 10.1073/pnas.1908513117

**Published:** 2019-12-23

**Authors:** Xiaoqi Zheng, Yonglong Lu, Jingjing Yuan, Yvette Baninla, Sheng Zhang, Nils Chr. Stenseth, Dag O. Hessen, Hanqin Tian, Michael Obersteiner, Deliang Chen

**Affiliations:** ^a^State Key Laboratory of Urban and Regional Ecology, Research Centre for Eco-Environmental Sciences, Chinese Academy of Sciences, 100085 Beijing, China;; ^b^School of Environment & Natural Resources, Renmin University of China, 100872 Beijing, China;; ^c^Key Laboratory of the Ministry of Education for Coastal Wetland Ecosystems, College of the Environment and Ecology, Xiamen University, 361102 Fujian, China;; ^d^University of Chinese Academy of Sciences, 100049 Beijing, China;; ^e^Centre for Ecological and Evolutionary Synthesis, University of Oslo, 03160 Oslo 3, Norway;; ^f^Ministry of Education Key Laboratory for Earth System Modeling, Department of Earth System Science, Tsinghua University, 100084 Beijing, China;; ^g^Section for Aquatic Biology and Toxicology, Centre for Biogeochemistry in the Anthropocene, University of Oslo, 03160 Oslo 3, Norway;; ^h^International Center for Climate and Global Change Research, Auburn University, Auburn, AL 36849;; ^i^School of Forestry and Wildlife Sciences, Auburn University, Auburn, AL 36849;; ^j^Ecosystem Services and Management Program, International Institute for Applied Systems Analysis, A-2361 Laxenburg, Austria;; ^k^Regional Climate Group, Department of Earth Sciences, University of Gothenburg, 405 30 Gothenburg, Sweden

**Keywords:** CO_2_ emissions, energy consumption, policy change

## Abstract

CO_2_ emissions are of global concern because of climate change. China has become the largest CO_2_ emitter in the world and presently accounts for 30% of global emissions. Here, we analyze the major drivers of energy-related CO_2_ emissions in China from 1978 when the reform and opening-up policy was launched. We find that 1) there has been a 6-fold increase in energy-related CO_2_ emissions, which was driven primarily (176%) by economic growth followed by population growth (16%), while the effects of energy intensity (−79%) and carbon intensity (−13%) slowed the growth of carbon emissions over most of this period; 2) energy-related CO_2_ emissions are positively related to per capita gross domestic product (GDP), population growth rate, carbon intensity, and energy intensity; and 3) a portfolio of command-and-control policies affecting the drivers has altered the total emission trend. However, given the major role of China in global climate change mitigation, significant future reductions in China’s CO_2_ emissions will require transformation toward low-carbon energy systems.

The largest absolute national contribution to global CO_2_ emissions now is from China (1, 2), which currently accounts for ∼30% of global emissions (3). Like many other countries, the primary cause of anthropogenic CO_2_ emissions is energy-related fossil fuel combustion (4). China’s economy has increased rapidly, with an annual growth rate of 9.4% from 1978 to 2018 (5). There is a strong coupling relationship between the per capita gross domestic product (PCG) and the energy use per capita for China (*SI Appendix*, Fig. S1). A very strong positive correlation has been observed between the PCG and the energy consumption per capita (*SI Appendix*, Table S1). China’s energy consumption has increased along with the economy from 400 million tons of oil equivalent (Mtoe) in 1978 to 3,248 Mtoe in 2018, with an annual growth rate of 5.4% (6). The increasing energy consumption has had a significant negative impact on China’s environment in terms of land use change; pollution of air, water, and soil; and biodiversity loss on land and in the ocean (7–9); it has also resulted in significant CO_2_ emissions, thereby strongly increasing the relative contribution from China to the global atmospheric CO_2_ concentration (10). There is also a strong coupling relationship between PCG and CO_2_ emissions per capita in China (Fig. 1*A*), and it has been demonstrated that the economic growth remains strongly coupled with CO_2_ emissions (11).

**Fig. 1.**
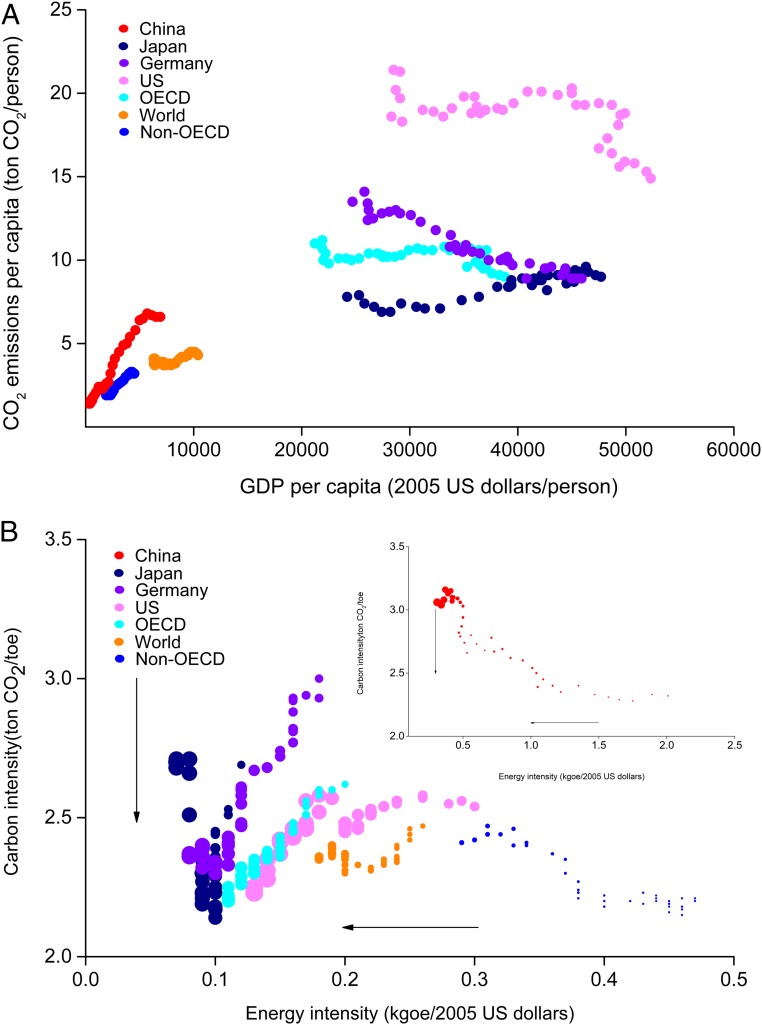
Dynamic changes in the key indexes in major countries or regions between 1978 and 2016. *A* shows the CO_2_ emissions per capita change along with GDP per capita; the trajectories are moving from left to right. In *B*, the EI is on the *x* axis, the CI is on the *y* axis, and the bubble size represents the PCG; the trajectories are moving from right to left and from top to bottom. The data are from the IEA (3).

The Chinese authorities recognize China’s role in global CO_2_ emissions reduction and climate change mitigation and have accordingly strengthened their efforts to combat climate change: for example, by launching a “revolutionary strategy for energy production and consumption.” This strategy is aimed at enhancing energy conservation, optimizing the energy structure, and supporting the development of nuclear energy and renewables (12). With these efforts, the increase in energy-related CO_2_ emissions has been reduced. However, future emissions still need to be decreased significantly, and the drivers of emissions per se as well as the political and technological drivers of the reduced total and relative (per capita or gross domestic product [GDP]) CO_2_ emissions need to be explored, quantified, and better understood.

We applied the Kaya identity method to analyze the observed trends. This analytical tool allocates the contribution of the change in CO_2_ emissions into the product of 4 factors, namely population size (P), PCG, energy intensity (EI) per unit of GDP, and emission per unit of energy consumed (carbon intensity [CI]) (13). The contribution of each of these factors to CO_2_ emissions can be assessed by index decomposition analysis (IDA) (14–17). To the best of our knowledge, the major determinants over the past 40 y have not been fully identified and quantitatively assessed. Temporal trends in carbon emissions in China have only been analyzed over brief time spans (18–22), and therefore, we extended this study to a timescale of 40 y. Although China’s CO_2_ emissions have continued to rise, they have not increased at the same rate over time. Thus, analyzing the 4 factors that have affected CO_2_ emissions from 1978 to 2018 can help policy makers and stakeholders to understand the historical changes and determine how to curb increases in CO_2_. We applied the logarithmic mean divisia index (LMDI) method (15), which is a commonly used IDA approach, with the help of the Kaya identity and econometric analysis method to quantitatively assess the determinants driving China’s CO_2_ emissions growth since 1978. Details of the Kaya identity, LMDI methods, and data sources are provided in *Methods*.

## Results

### Dynamic Changes in China’s Energy-Related CO_2_ Emissions.

An accurate account of energy-related emissions in China from 1978 to 2018 is the natural point of departure for a decomposition analysis. However, annual official reports on CO_2_ emissions have not been published by the Chinese authorities, with the exception of the national CO_2_ emission inventories in 1994, 2005, and 2012 (23). Nevertheless, several international organizations and databases, such as the International Energy Agency (IEA), the Emission Database for Global Atmospheric Research (EDGAR), the Carbon Dioxide Information Analysis Center, and the Climate Access Indicators Tool, as well as British Petroleum (BP) have published reports on China’s CO_2_ emissions, which were used in this study. We also calculated the national CO_2_ emissions based on official energy consumption data and established emission factors (*Methods*).

**Fig. 2.**
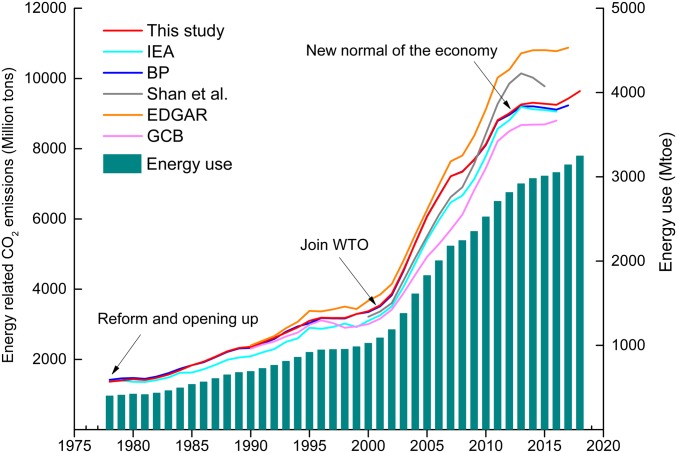
Energy-related CO_2_ emissions in China from 1978 to 2018. The *y* axis plots the energy-related CO_2_ emissions, referring to the carbon dioxide emissions emitted from the fossil fuel combustion process. Not all of the datasets with CO_2_ emissions started in 1978; Shan et al. (23) calculated 2 series of CO_2_ emissions. Here, we selected the reference CO_2_ emissions (gray line). Data sources are this study (5), IEA (3), BP (25), Shan et al. (23), EDGAR (26), global carbon project (GCB) (27), and energy use (6).

A comparison between the data obtained from international databases and China’s official data from emission inventories showed that the IEA data had the best match (24). However, the CO_2_ emissions calculated in the current work (red curve in Fig. 2) matched well with those of BP. To this end, China’s CO_2_ emissions have increased rapidly from 1.37 Gt CO_2_ in 1978 to 9.64 Gt CO_2_ in 2018, with an average annual growth of 5.0%. The emission history can be roughly divided into 3 stages. The first stage began with the launch of reform and opening up and ended in 2000, during which time the CO_2_ emissions increased slowly at an average annual rate of 4.2%. The second stage started in 2001 when China joined the World Trade Organization (WTO) and ended in 2012, which had an average annual growth rate of 8.5%. The third stage was post-2012 when China’s economy entered a “new normal.” The growth of CO_2_ emissions slowed to an average annual growth rate of 0.81% between 2013 and 2018.

### Four Drivers of CO_2_ Emissions.

**Fig. 3.**
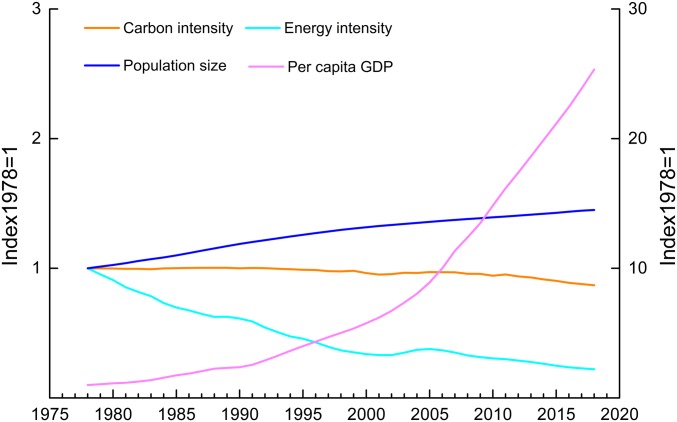
Historical changes in the 4 indicators from 1978 to 2018; 1978 was used as the base year, and therefore, the value of the indicator in 1978 was set as 1. CI (orange), EI (cyan), and population size (blue) are shown on the left *y* axis, while PCG (purple) is shown on the right *y* axis.

The amount of annual CO_2_ emission was calculated as the product of the CI, EI, PCG, and P. However, the historical changes in these 4 indicators were very different (Fig. 3; absolute numbers of the 4 indicators are listed in *SI Appendix*, Table S2). The CI declined by only 13.1% compared with that in 1978, despite efforts to optimize the energy structure. This was due to the high proportion of coal in primary energy consumption, which still constituted 59.0% in 2018 (11.7 percentage points lower than that in 1978). With the continuous improvement of economic and technical efficiencies, the EI also continued to decline. In 2018, the EI had decreased by 77.9% compared with that in 1978 but was still higher than the EI of major developed countries (Fig. 1*B*). The PCG displayed a significantly higher growth rate than population growth, and the PCG in 2018 was more than 25 times greater than that in 1978. China has implemented a family planning policy since 1978, and the population growth rate (PR) has been effectively controlled. In 2018, the total population had increased by 45.0% compared with that in 1978, while the CO_2_ emission per capita had increased by 387%.

### Decomposition Analysis through the Lens of Policy Changes.

By evaluating the relationship between macroeconomic policy change and carbon emissions during each 5-y planning period, it seemed plausible that national macroeconomic policies have affected the trend of carbon emissions by affecting the 4 drivers in different ways over time. The changes in emission regimes and contributions from the various contributors were closely linked to national policy programs over the various periods (Table 1). For instance, in 1978 the Chinese government began to comprehensively adjust the development direction of the national economy from a planned economy to a socialist market economy. As a result, China’s economic efficiency gradually improved from the restrictive economic system affected by the Cultural Revolution (11). During the first 3 y of reform (1978 to 1980), the economic growth rate was significantly higher than the growth rate of energy consumption. This caused the decline of both the EI and the CI. The reform and opening-up policy also increased domestic demand, leading to rapid economic growth and increasing CO_2_ emissions. Another example was China joining the WTO, which marked the start of the second stage of increase in CO_2_ emissions. After China joined the WTO in 2001, the international market was open to China without restriction, and investment was increased in energy-intensive industrial production, which led to a sharp increase in energy consumption and a significant rebound in EI (28). The effects of all of the 4 indicators were positive in the tenth 5-y plan (FYP) period (2001 to 2005).

**Table 1.** Key policies and cumulative determinant effects from 1978 to 2018 (million tons)

Year	Policies or events	Net effect	CI effect	EI effect	PCG effect	P effect
1978–1980 (5th)	1) Started reform and opening up; 2) started socialist market economy; 3) implemented executive orders on control over energy use; 4) launched family planning policy	73	−2	−133	173	35
1981–1985 (6th)	1) Implemented household land contract-responsibility system; 2) established energy conservation management system	396	5	−413	695	109
1986–1990 (7th)	1) Dual-pricing system-driven inflation; 2) political turmoil; 3) enacted interim regulations on energy conservation management	525	−3	−267	631	164
1991–1995 (8th)	1) Strengthened reform and opening-up policy in 1992; 2) started to establish energy-saving standards	739	−34	−790	1,409	155
1996–2000 (9th)	1) Shut down 15 major categories of small heavy-pollution enterprises; 2) enacted Energy Conservation Law; 3) Southeast Asia financial crisis and severe flood disasters	274	−86	−968	1,182	146
2001–2005 (10th)	1) China joined the WTO; 2) Enacted medium- and long-term special plans for energy conservation; 3) launched Population and Family Planning Law	2,692	50	557	1,949	135
2006–2010 (11th)	1) Launched a policy package to expand domestic demand, addressing the global financial crisis; 2) listed EI as binding target for the FYP; 3) addressed Copenhagen pledge	2,063	−221	−1,540	3,644	181
2010–2015 (12th)	1) China’s economy enters new normal stage; 2) 12th FYP for energy conservation and emission reduction; 3) implemented national plan on climate change (2014–2020); 4) work plan for controlling greenhouse gas emissions during 12th FYP; 5) launched 2 children per household policy	1,150	−403	−1,853	3,183	224
2016–2018 (13th)	1) Launched supply-side structural reform; 2) 13th FYP for energy conservation and emission reduction; 3) enhanced actions on climate change: China’s nationally determined contributions; 4) work plan for controlling greenhouse gas emissions during 13th FYP	362	−356	−1,115	1,693	140

China has released an FYP for national economic and social development every 5 y. The annual decomposition results for the 4 indicators are provided in *SI Appendix*, Table S3. The cumulative determinant effects reflect the performances of the macroeconomic policies launched in the FYPs.

### Further Analysis in Aggregate.

**Fig. 4.**
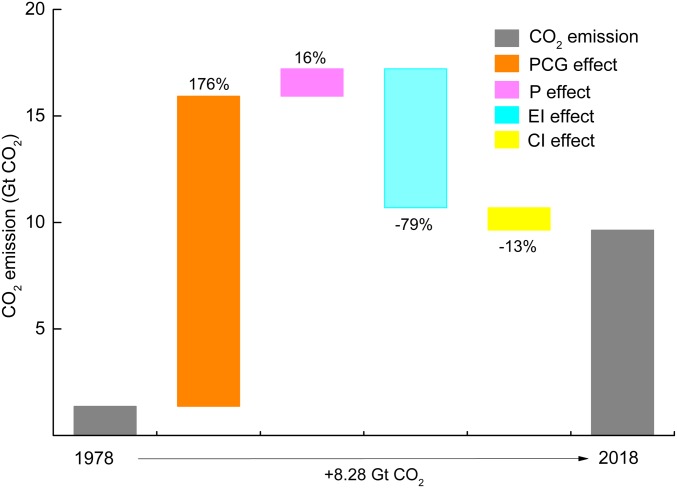
Cumulative determinant effects of 4 indicators from 1978 to 2018. The percentages above the *y* axis refer to the determinant’s contributions to the changes in CO_2_ emissions.

Temporal changes in the positive and negative contributions from the different drivers (*SI Appendix*, Table S3) revealed policy-driven dynamics. The PCG and P contributed significantly to carbon emissions over the entire period, while the EI and CI effects shifted between positive and negative and had a downward influence on CO_2_ emissions for most years. China’s CO_2_ emissions have increased since 1978 and increased by 6-fold between 1978 and 2018. Looking at this time span in aggregate (Fig. 4), the most significant contributor to increased CO_2_ emissions was PCG, which contributed 176% of the overall change in CO_2_ emissions between 1978 and 2018. The second most important factor was population growth, which contributed 16% of the overall change. The EI and CI effects displayed the opposite trend and caused reductions in CO_2_ emissions by 79 and 13%, respectively.

We also conducted an econometric analysis to further validate the relationships between CO_2_ emissions and the 4 driving factors. The Johansen cointegration test demonstrated that there was a long and stable cointegration between China’s CO_2_ emissions and the CI, EI, PCG, and PR, with elasticity coefficients for the 4 variables of 2.24, 1.16, 1.25, and 0.50, respectively, thereby implying that they all had a positive correlation with CO_2_ emissions. For example, when PCG, EI, and PR were kept unchanged, if the CI was reduced by 1%, then China’s CO_2_ emissions would be decreased by 2.24%; when CI, EI, and PR were kept unchanged, if the PCG was increased by 1%, then China’s CO_2_ emissions would be increased by 1.25%. Johansen test not only verified the positive or negative effects of 4 indicators that concluded from LMDI method but also found that the CI had the largest potential influence on changes in CO_2_ emissions between the 4 indicators. However, in reality, CI has the smaller contribution to slow the increase in CO_2_ emissions compared with EI. The small contribution of carbon emission intensity was mainly due to the persistent high proportion of fossil fuel consumption, especially coal consumption. The Chinese government has strongly aimed at reducing EI since 1978, while the decrease of CI was begun from the implementation of Copenhagen pledge in the 11th FYP (2006 to 2008).

**Fig. 5.**
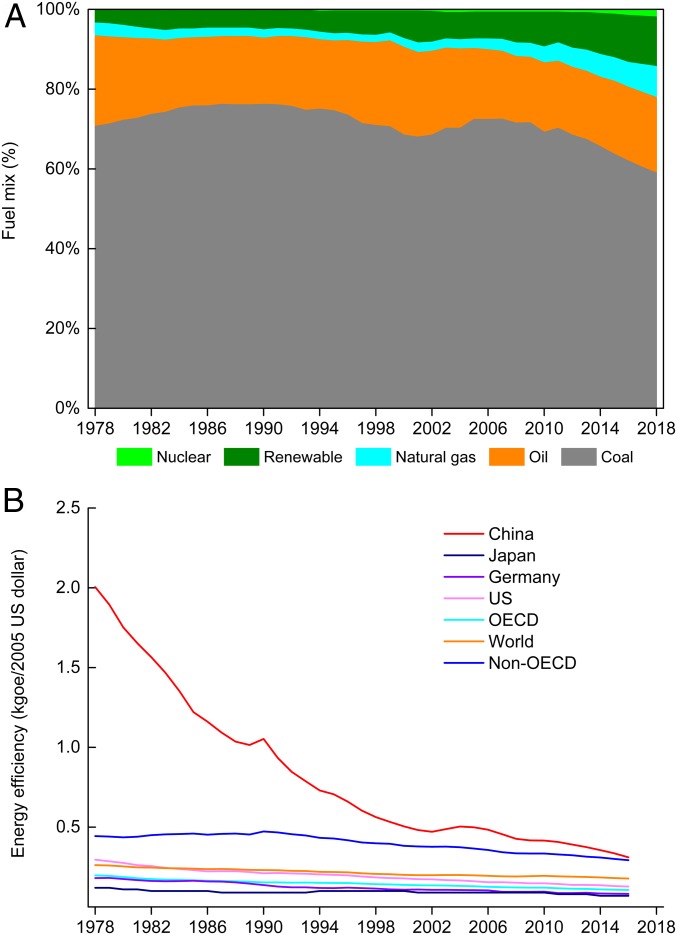
Dynamic changes in the fuel mix and energy efficiency. *A* shows fuel mix changes in primary energy consumption in China between 1978 and 2018. *B* presents comparison of energy efficiency between China and major developed countries between 1978 and 2016; the energy efficiency of major countries was calculated from the data from the IEA (3).

Although the CI reduction started later, the Chinese government has implemented ambitious policies to promote nonfossil fuel energy, and the decline in CI is increasingly contributing to lower carbon emissions. Furthermore, the installed capacity of renewable energies, such as wind, hydro, and solar powers, is the largest in the world. The proportion of nonfossil fuel energy in primary energy consumption increased from 3.4% in 1978 to 14.3% in 2018, which was 0.5 percentage points lower than that of the global average level in 2017. Even though the fuel mix was slightly optimized (e.g., with coal and oil decreasing, gas and renewables increasing, and nuclear increasing by a small percentage), the proportion of coal consumption decreased from 70.7% in 1978 to 59.0% in 2018, which was 31.4 percentage points above the global average level in 2017 (Fig. 5*A*). Although predictions for China’s energy use vary between different research institutes, there is a consensus that China’s energy use will continue rising until 2030 and then peak between 2030 and 2050. However, the fuel mix will continue being optimized until 2050 because of the low-carbon development trend (29–32).

The comparison of energy efficiency between China and some developed countries, such as the United States and Japan, shows that China has made significant progress over the last 40 y but still lags behind developed countries. As such, there is room for China to further improve its energy efficiency (Fig. 5*B*). For example, the energy system efficiency of China increased from 25.9% in 1980 to 36.1% in 2012, while the energy system efficiency of Organization for Economic Cooperation and Development (OECD) countries has been more than 41.0% since the early 1990s (33). Energy efficiency improvement was evident since 1978, and our analysis demonstrated that China’s opening-up policy as well as the adjustment of its industrial structure and reform of its economic system were major contributors to this trend (34). Further improvement of energy efficiency may originate from 2 drivers, namely technological improvement and industrial structure optimization (29). On one hand, energy efficiency from improvement in technology will gradually decrease because the energy consumption per unit of product for major energy-intensive industries has already been improving steadily over the past 40 y. For example, the EI of crude steel production in 2015 was 644 kgce/t, while the global advanced level was 602 kgce/t (6). On the other hand, China’s economic development still relies on energy-intensive industries, and the share of its secondary industry is significantly higher than that in other major developed countries. In 2017, the total energy use of 6 energy-intensive industries* accounted for 81.8% of the total industrial energy consumption (6); however, the added value of 6 energy-intensive industries accounted for only 29.7% of the total added value of industries (35). This means that the optimization of industrial structures will be important to reduce the EI in the future.

## Discussion and Policy Implications

Our analysis suggested that, since the launch of the reform and opening-up policy, China has enacted, reinforced, or adjusted economic policies to satisfy developmental and environmental needs. Subsequently, the PCG and P have increased steadily with different growth rates, while the EI and CI have decreased but with some variability. Finally, these 4 drivers together influence the dynamic trend of China’s CO_2_ emissions. In addition, our analysis may be helpful in assessing the long-term trends and goals of CO_2_ emissions. The Chinese government pledged to continue with economic reform and an open market at the 40th anniversary of China’s reform and opening-up policy (36). This means that the policy environment for economic growth and energy efficiency improvement will exist for a long time, and a medium-high GDP growth rate is both possible and feasible (37–39). A less strict family planning policy did not trigger an increase in the fertility rate, but the adjustment of the family planning policy can boost labor resources and delay the process of population aging (40). In this context, many research institutes have predicted that China's population will continue to grow until at least 2030 (29, 30, 41). Thereby, economic growth and population size will remain, driving the increase in CO_2_ emission in the long term.

There is still a significant gap in energy efficiency between China and developed countries. Thus, more attention should be paid to improving the efficiency of energy-intensive industries. China currently prioritizes energy conservation, energy saving, and emissions reduction, and this will eventually promote a downward trend in CO_2_ emissions. China has also formulated and started to implement a more proactive policy on renewable energy development. The development of renewable energy will gradually accelerate, and the energy structure will also be further improved. It is expected that, by 2030 and 2050, the proportions of nonfossil fuel energy consumption will reach 20 and 50%, respectively (42).

Based on the discussion above, energy-related CO_2_ emissions will continue to increase in the near future, but the growth rate of CO_2_ emissions should remain at a relatively low level. Considering the 2030 CO_2_ emissions peak target and the responsibility of helping the world achieve the Paris climate targets, the next 10 to 15 y will be critical for China to reach the carbon emission peak (29). Thus, China must make a transition toward a low-carbon economy and low-carbon energy systems and improve consumption behavior under the ongoing policy of reform and opening up.

First, the effective way to control CO_2_ emissions is to promote the low-carbon transformation of economic growth (e.g., decoupling, which is where economic development occurs with less energy consumption). This strives to achieve long-term, high-quality development by optimizing the economic structure and transforming the drivers of economic growth. This includes strict control over the growth of energy-intensive industries as well as upgrading traditional industries through technological innovation and developing emerging industries that consume less energy. Furthermore, these measures can also help decrease the EI.

Second, a low-carbon transformation of energy systems should be implemented to improve energy efficiency and optimize the energy structure. Improving energy efficiency and increasing the share of renewable energy play a key role in decarbonizing the energy system around 2050 all over the world (43), especially in China. Regarding energy efficiency improvement, higher energy efficiency standards in the industry, building, and transportation sectors should be formulated and implemented. These standards would explore the energy conservation potential through economic structure optimization and energy management. With regard to optimizing energy structure, strong economic incentives to shift from fossil fuels to renewable energy will help to replace fossil fuels with nonfossil fuel alternatives in the dynamics of the energy transition. On one hand, it is important to strive for a fast reduction in coal consumption by setting a coal consumption cap and developing clean coal production and utilization technologies, such as coal cleaning technologies and carbon capture and storage technologies. On the other hand, it is imperative to speed up the development of nonfossil fuels, especially renewables, by increasing renewable investment, setting more active and binding goals, giving priority to nonfossil fuels in gaining grid access, and establishing a carbon emission trade market (44).

Third, it is a public endeavor to change consumption behaviors to reduce the per capita energy consumption by advocating a low-carbon consumption culture throughout society. Currently, the per capita energy use in China is much lower than that of the United States or other developed countries (*SI Appendix*, Fig. S1). However, if Chinese consumers consumed energy in the same manner as American consumers, then the energy use per capita would increase 3-fold, thereby causing a corresponding increase in CO_2_ emissions and offsetting the mitigation by EI and CI improvements. Thus, the government should not only cultivate a low-carbon consumption culture but also, make efforts to increase investments in the construction of a low-carbon infrastructure that includes waste recycling stations, charging stations, and environmentally friendly public transit systems (45). Investment in afforestation should also be reinforced. The current greening in China, of which 42% is related to enhanced forest growth, is an important contributor to CO_2_ sequestration and the mitigation of climate change (46). Moreover, enterprises should focus on improving consumer satisfaction with cost-effective, low-carbon products to satisfy consumer demand. Finally, the public needs to shift from environmentally damaging and unhealthy consumption behaviors to a green and low-carbon behaviors through policy interventions (47).

## Methods

### Approach for Calculating CO_2_ Emissions.

The reference approach is a top-down approach using the nation’s energy consumption data to calculate CO_2_ emissions from fossil fuel combustion when primary energy consumption data are easily available (23). We calculated the energy-related CO_2_ emissions using Eq. **1**, and only 3 types of primary fossil fuels (coal, petroleum, and natural gas) were considered:

$$CO_{2}=\sum AD_{i}\times EF_{i},$$ [1]

where *CO*_2_ refers to the national or regional CO_2_ emissions of China and *AD*_*i*_ and *EF*_*i*_ are the primary energy consumption and emission factors of fossil fuels, respectively.

### Kaya Identity.

The Kaya identity shows that the national or regional CO_2_ emissions are equal to the product of 4 indicators, namely CI, EI, PCG, and P. The Kaya identity was first proposed by Japanese scholar Yoichi Kaya at an Intergovernmental Panel on Climate Change (IPCC) seminar (13). The formula illustrates a relationship between macroeconomic indicators, such as CI, EI, P, and GDP. The 4 indicators in the formula cannot only be used to assess the determinants driving China’s CO_2_ emission growth but also, provide useful perspectives to review policy changes in the long run. The formula can be expressed as Eq. **2**:

$$CO_{2}=\frac{CO_{2}}{E}\times \frac{E}{GDP}\times \frac{GDP}{P}\times P=CI\times EI\times PCG\times P,$$ [2]

where *E* refers to the primary energy consumption of China, *GDP* refers to the GDP of China, and P refers to population size. $\frac{CO_{2}}{E}$ refers to the CI, $\frac{E}{GDP}$ refers to EI, and $\frac{GDP}{P}$ refers to the PCG.

### LMDI Method.

We adopted the LMDI method to assess the contribution of different indicators to the overall change in the energy-related CO_2_ emissions in China from 1978 to 2018. The LMDI method has either an additive or multiplicative mathematical form, but the final decomposition results of the 2 forms are the same (14, 48). This study adopted the additive form, which decomposes the difference of the indicator form between time *t* and *t* − 1 into a number of determinant effects. We adopted the Kaya identity to separate the influences of the 4 indicators on the overall change in the CO_2_ emissions according to Eq. **2**. According to the definition of the additive form of the LMDI method, the difference in China’s carbon emissions between 2 adjacent years (Δ*CO*_2_) is equal to the sum of the impacts from individual indicators, namely CI (Δ*CI*), EI (Δ*EI*), PCG (Δ*PCG*), and P (Δ*P*), as described in Eq. **3**. For convenience, Δ*CO*_2_, Δ*CI*, Δ*EI*, Δ*PCG*, and Δ*P* were defined as net effect, CI effect, EI effect, PCG effect, and P effect, respectively:

$$\Delta CO_{2}=CO_{2}\left(t\right)-CO_{2}\left(t-1\right)=\Delta CI+\Delta EI+\Delta PCG+\Delta P.$$ [3]

The relative contribution of each indicator was calculated separately by Eqs. **4** to **7**. If the Δ*CI* is a positive value, then the CI effect is positive and promotes carbon emission growth; if the Δ*CI* is a negative value, then the CI effect is negative and slows carbon emission growth. The same rule applies to the EI effect, the PCG effect, and the P effect:

$$\Delta CI=\sum \frac{CO_{2}\left(t\right)-CO_{2}\left(t-1\right)}{\text{ln}CO_{2}\left(t\right)-\text{ln}CO_{2}\left(t-1\right)}\times \text{ln}\left(\frac{CI\left(t\right)}{CI\left(t-1\right)}\right)$$ [4]

$$\Delta EI=\sum \frac{CO_{2}\left(t\right)-CO_{2}\left(t-1\right)}{\text{ln}CO_{2}\left(t\right)-\text{ln}CO_{2}\left(t-1\right)}\times \text{ln}\left(\frac{EI\left(t\right)}{EI\left(t-1\right)}\right)$$ [5]

$$\Delta GPC=\sum \frac{CO_{2}\left(t\right)-CO_{2}\left(t-1\right)}{\text{ln}CO_{2}\left(t\right)-\text{ln}CO_{2}\left(t-1\right)}\times \text{ln}\left(\frac{PGC\left(t\right)}{PCG\left(t-1\right)}\right)$$ [6]

$$\Delta P=\sum \frac{CO_{2}\left(t\right)-CO_{2}\left(t-1\right)}{\text{ln}CO_{2}\left(t\right)-\text{ln}CO_{2}\left(t-1\right)}\times \text{ln}\left(\frac{P\left(t\right)}{P\left(t-1\right)}\right).$$ [7]

### Data Sources.

The GDP and population size data were taken from the China Statistical Yearbooks, and the nominal GDP in different years was adjusted to the GDP at the 2010 constant price by the GDP index. The primary energy consumption and its structure were compiled from the China Energy Statistical Yearbooks. We used 2.64, 2.08, and 1.63 tCO_2_/tce as the carbon emission factors of coal, petroleum, and natural gas, respectively. These emission factors were calculated according to the first biennial update report on the climate change of China (49).

### Johansen Cointegration Test.

The data on CO_2_ emissions, CI, EI, PCG, and P were of time series nature. In order to avoid multicollinearity, the indicator P was replaced by PR. A stationarity test should be applied to the time series data to prevent spurious regression or invalidation of the *t* test (50). If the time series data are nonstationary, the ordinary least square (OLS) method cannot be conducted to identify relationship between these variables because the prerequisite for OLS method is that time series data should be stationary. However, cointegration test was developed to identify relationships between nonstationary time series variables, and Johansen cointegration test, one kind of cointegration test, can be used to identify cointegration relationship between at least 3 time series variables. This study utilized the augmented Dickey–Fuller (ADF) test to identify whether CO_2_ emissions (expressed as *CE*), CI, EI, PCG, and PR were stationary or not. The ADF test has a null hypothesis of a unit root against the alternative of no unit root (51). All of the variables were transformed through the natural logarithm to eliminate possible heteroscedasticity before the ADF test. The results of the ADF test (*SI Appendix*, Table S4) illustrated that all 5 variables (ln*CE*, ln*CI*, ln*EI*, ln*PCG*, and ln*PR*) were integrated of order 2, meaning that they are cointegrated. A long equilibrium relationship existed between these 5 variables, which could be characterized as Eq. **8**:

$$\ln\;CE=\alpha +\beta_{1}\;\ln\;CI+\beta_{2}\;\ln\;EI+\beta_{3}\;\ln\;PCG+\beta_{4}\;\ln\;PR+\varepsilon ,$$ [8]

where α is a constant; $\beta_{1}$, $\beta_{2}$, $\beta_{3}$, and $\beta_{4}$ are the elasticity coefficients of CE to CI, EI, PCG, and PR, respectively; and ε is a stochastic disturbance term.

The Johansen test was used to identify the cointegration relationship. Before applying the Johansen test, the optimal lag of the unrestricted vector autoregressive (VAR) model should be selected. The optimal lag can be selected according to information criteria, such as the final prediction error criterion, Akaike information criterion, Schwarz information criterion, and Hannan–Quinn information criterion (50). According to the comparison of the results of the information criteria (*SI Appendix*, Table S5), the optimal lag for the unrestricted VAR was selected as 2. Thus, the lag for the Johansen test was determined to be 1. Then, the maximum eigenvalue test was applied to check the cointegration rank, which determines the numbers of the cointegration equation (50). The maximum eigenvalue test result (*SI Appendix*, Table S6) showed that the null hypothesis that there was no cointegration relationship was rejected, indicating 1 cointegrating equation at the 0.05 significance level. Finally, the long-term equilibrium cointegrating equation can be expressed as follows:

ln CE=2.24 ln CI+1.16 ln EI+1.25 ln PCG+0.50 ln PR+3.78. [9]

### Limitations.

Because of limited data availability, we calculated the emission factors from the first biennial update report on the climate change of China and used them for all years. The factors may have been different from year to year. Furthermore, we only considered energy-related CO_2_ emissions, while the industrial process-related CO_2_ emissions, such as those from cement production, were not considered. Therefore, further research is needed to extend the coverage of CO_2_ emissions.

### Data and Materials Availability.

All data are available in the main text or *SI Appendix*.

## Supplementary Material

Supplementary File

## Footnotes

*Six energy-intensive industries are 1) smelting and pressing of ferrous metals; 2) manufacturing of raw chemical materials and chemical products; 3) manufacturing of nonmetallic mineral products; 4) processing of petroleum and coking and processing of nuclear fuel; 5) smelting and pressing of nonferrous metals; and 6) producing and supplying of electric power and heat power.
