# Supplementary material for: Drivers of change in China’s energy-related CO2 emissions
Source: Proc Natl Acad Sci U S A. 2019 Dec 23;117(1):29–36. doi: 10.1073/pnas.1908513117 (PMC6955364; doi:10.1073/pnas.1908513117)
Supplement: Supplementary File [file pnas.1908513117.sapp.pdf]

Supplementary material

Drivers of change in China's energy-related CO_2 emissions

Xiaoqi Zheng[a,b], Yonglong Lu[a,c,d,1], Jingjing Yuan[a], Yvette Baninla[a,d], Sheng Zhang[a,b], Nils Chr. Stenseth[e,f,1], Dag O. Hessen[g], Hanqin Tian[a,h,i], Michael Obersteiner[j], Deliang Chen[k]

[a]State Key Laboratory of Urban and Regional Ecology, Research Centre for Eco-Environmental Sciences, Chinese Academy of Sciences, Beijing 100085, China.

[b]School of Environment & Natural Resources, Renmin University of China, Beijing 100872, China.

[c]Key Laboratory of the Ministry of Education for Coastal Wetland Ecosystems, College of the Environment and Ecology, Xiamen University, Fujian 361102, China.

[d]University of Chinese Academy of Sciences, Beijing 100049, China.

[e]Centre for Ecological and Evolutionary Synthesis, University of Oslo, 03160 Oslo 3, Norway.

[f]Ministry of Education Key Laboratory for Earth System Modeling, Department of Earth System Science, Tsinghua University, Beijing 100084, China.

[g]Section for Aquatic Biology and Toxicology, Centre for Biogeochemistry in the Anthropocene, University of Oslo, 03160 Oslo 3, Norway.

[h]International Center for Climate and Global Change Research, Auburn University, Auburn, AL 36849, USA.

[i]School of Forestry and Wildlife Sciences, Auburn University, Auburn, AL 36849, USA.

[j]Ecosystem Services and Management Program, International Institute for Applied Systems Analysis, A-2361, Laxenburg, Austria.

[k]Regional Climate Group, Department of Earth Sciences, University of Gothenburg, 405 30 Gothenburg, Sweden.

[1]To whom correspondence maybe addressed. Email: yllu@rcees.ac.cn; n.c.stenseth@ibv.uio.no.

This file includes:

Figure. S1, S2
Table. S1 to S6
Decomposition analysis in terms of the Five-Year Plans

www.pnas.org/cgi/doi/10.1073/pnas.1908513116

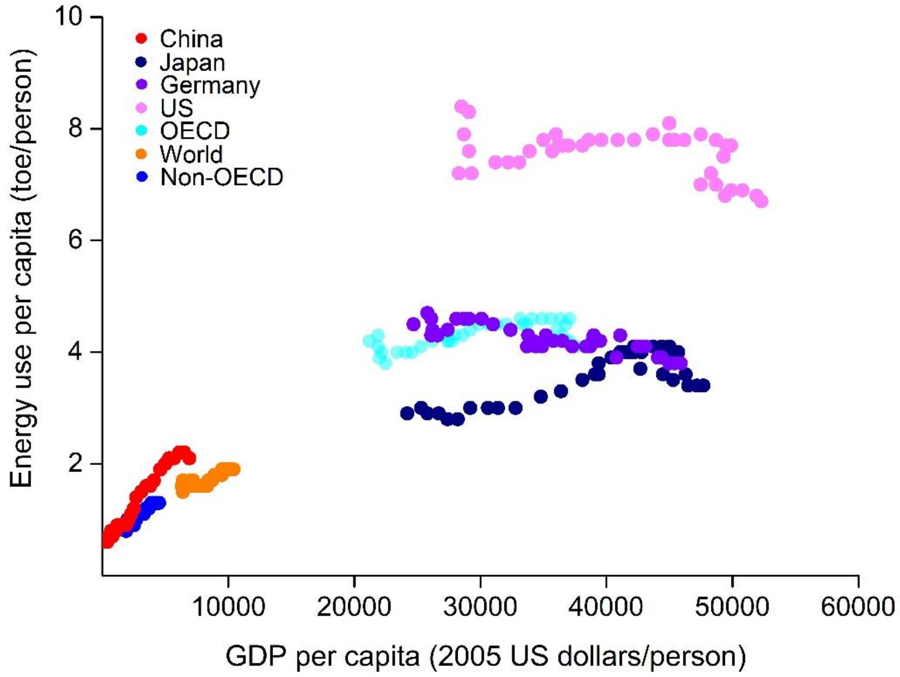

Figure. S1 Dynamic changes in the energy use per capita along with GDP per capita between 1978 and 2016

Table S1 Correlation coefficients between per capita GDP and per capita index in major countries

Per capita index	China	US	Japan	Germany	World	OECD	Non-OECD
Per capita Energy use	0.988 **	-0.420 **	0.802 **	-0.858 **	0.871 **	0.555 **	0.972 **
Per capita CO_2	0.986 **	-0.602 **	0.891 **	-0.966 **	0.792 **	-0.356 **	0.984 **

Note: Two asterisks mean extremely significant differences (P<0.01).

Table S2 Trends for key indexes of China from 1978 to 2018

Year	Energy use	Coal	Oil	Natural gas	Non-fossil	GDP	Population	CO_2 emissions	Per capita GDP	Carbon intensity	Energy intensity
	Mtoe	%	%	%	%	Million RMB	Million	Million ton CO_2	10^4 RMB per capita	ton CO_2/ 10^4 RMB	toe / 10^4 RMB
1978	400	70.7	22.7	3.2	3.4	200	963	1366	0.21	6.84	2.00
1979	410	71.3	21.8	3.3	3.6	215	975	1400	0.22	6.51	1.91
1980	422	72.2	20.7	3.1	4.0	232	987	1439	0.23	6.21	1.82
1981	416	72.7	20.0	2.8	4.5	243	1001	1415	0.24	5.81	1.71
1982	434	73.7	18.9	2.5	4.9	265	1017	1477	0.26	5.56	1.64
1983	462	74.2	18.1	2.4	5.3	294	1030	1568	0.29	5.33	1.57
1984	496	75.3	17.4	2.4	4.9	339	1044	1694	0.32	5.00	1.47
1985	537	75.8	17.1	2.2	4.9	384	1059	1835	0.36	4.78	1.40
1986	566	75.8	17.2	2.3	4.7	418	1075	1937	0.39	4.63	1.35
1987	606	76.2	17.0	2.1	4.7	467	1093	2079	0.43	4.45	1.30
1988	651	76.1	17.1	2.1	4.7	520	1110	2231	0.47	4.29	1.25
1989	679	76.1	17.1	2.1	4.7	541	1127	2325	0.48	4.29	1.25
1990	691	76.2	16.6	2.1	5.1	563	1143	2360	0.49	4.20	1.23
1991	726	76.1	17.1	2.0	4.8	615	1158	2488	0.53	4.05	1.18
1992	764	75.7	17.5	1.9	4.9	702	1172	2613	0.60	3.72	1.09
1993	812	74.7	18.2	1.9	5.2	800	1185	2763	0.67	3.45	1.02
1994	859	75.0	17.4	1.9	5.7	904	1199	2912	0.75	3.22	0.95
1995	918	74.6	17.5	1.8	6.1	1003	1211	3099	0.83	3.09	0.92

1996	946	73.5	18.7	1.8	6.0	1103	1224	3189	0.90	2.89	0.86
1997	951	71.4	20.4	1.8	6.4	1204	1236	3178	0.97	2.64	0.79
1998	953	70.9	20.8	1.8	6.5	1298	1248	3178	1.04	2.45	0.73
1999	984	70.6	21.5	2.0	5.9	1398	1258	3294	1.11	2.36	0.70
2000	1026	68.5	22.0	2.2	7.3	1517	1267	3374	1.20	2.22	0.68
2001	1089	68.0	21.2	2.4	8.4	1643	1276	3539	1.29	2.15	0.66
2002	1187	68.5	21.0	2.3	8.2	1792	1285	3871	1.40	2.16	0.66
2003	1380	70.2	20.1	2.3	7.4	1971	1292	4550	1.53	2.31	0.70
2004	1612	70.2	19.9	2.3	7.6	2170	1300	5307	1.67	2.45	0.74
2005	1830	72.4	17.8	2.4	7.4	2418	1308	6066	1.85	2.51	0.76
2006	2005	72.4	17.5	2.7	7.4	2725	1314	6644	2.07	2.44	0.74
2007	2180	72.5	17.0	3.0	7.5	3112	1321	7215	2.36	2.32	0.70
2008	2244	71.5	16.7	3.4	8.4	3414	1328	7343	2.57	2.15	0.66
2009	2353	71.6	16.4	3.5	8.5	3734	1335	7692	2.80	2.06	0.63
2010	2524	69.2	17.4	4.0	9.4	4130	1341	8129	3.08	1.97	0.61
2011	2709	70.2	16.8	4.6	8.4	4523	1347	8816	3.36	1.95	0.60
2012	2815	68.5	17.0	4.8	9.7	4880	1354	9009	3.60	1.85	0.58
2013	2918	67.4	17.1	5.3	10.2	5261	1361	9261	3.87	1.76	0.55
2014	2981	65.6	17.4	5.7	11.3	5645	1368	9311	4.13	1.65	0.53
2015	3009	63.7	18.3	5.9	12.1	6034	1375	9279	4.39	1.54	0.50
2016	3051	62.0	18.5	6.2	13.3	6438	1383	9251	4.66	1.44	0.47
2017	3143	60.4	18.8	7.0	13.8	6883	1390	9428	4.95	1.37	0.46
2018	3248	59.0	18.9	7.8	14.3	7337	1395	9641	5.26	1.31	0.44

Note: GDP in this table was calculated at 2010 constant price.

Decomposition analysis in terms of the Five-Year Plans.

The reform and opening-up policy was enacted by the Chinese government in 1978. Specific policies for boosting the economy, popularizing energy saving, optimizing the energy structure, or controlling population growth have been implemented successively since then. The cumulative net effects from 1978 to 1980 were 73 Mt of CO_2, of which the CI effects and EI effects were negative while the PCG effects and P effects were positive. On this basis, we assessed the changes that were implemented during the following eight FYPs.

The Sixth Five-Year Plan (1981–1985). During this period, only the EI effect was negative, while the effects of the other three indicators were positive. The major causes were as follows. Pursing economic development became a central societal task, and the energy used for economic development was gradually strengthened with an annual average GDP growth rate of 10.7% (1). Furthermore, the household-contract-responsibility-system policy liberated thousands of farmers from land and transferred the abundant human resources into secondary and tertiary industries (3). The secondary industries played a more important role in stimulating economic growth, and coal was the preferred energy source supporting industrial development. Thus, the proportion of coal consumption increased by 3.1 percentage points (2), and eventually led to a continuous decline in EI and a slight increase in CI. Furthermore, the family planning policy was designated as a national basic policy in 1982. As such, the P maintained a relatively low level of growth, which also enabled the P to promote the growth of carbon emissions. Therefore, the explanation for the P effect will not be described below.

The Seventh Five-Year Plan (1986–1990). The CI effect shifted from positive to negative, unlike that in the Sixth FYP period, while the effects of the other three indicators remained the same, with the EI effect being negative and the PCG effect and P effect being positive. The major reasons for this were as follows. In the first 2 years of the Seventh FYP period, economic development continued to maintain rapid growth similar to that of the Sixth FYP period. However, in the following 2 years, serious inflation and political turmoil occurred, and an unstable political and economic situation significantly decreased the economic growth rate (4), thereby leading to a decline in the annual average growth rate of 7.8% (1). Even so, the economic growth rate was still greater than the growth rate of energy consumption, and the proportion of coal consumption increased by only 0.4 percentage points (2). The relatively fast development of light industry

helped to optimize the industry structure, and both EI and CI decreased to some extent.

The Eighth Five-Year Plan (1991–1995). The CI and EI effects enhanced the negative effect on carbon emissions compared to the Seventh FYP period. The PCG effect also significantly strengthened the positive effect, while the P effect remained at the same positive level as that of the Seventh FYP period. The reasons for this were as follows. In the early stage of the Eighth FYP period, the social economy recovered from political turmoil and inflation. In 1992, the reform and opening-up policy was further strengthened by the government. Consequently, economic development accelerated again, infrastructure construction underwent rapid growth, the proportion of secondary industries increased by 5.4 percentage points (2), and the annual average growth rate of GDP reached 12% (1). Despite a significantly increased proportion of industrial added value, the change in industry structure greatly reduced the EI. The energy efficiency of the industrial sector also experienced a clear decrease. Nevertheless, the proportion of coal consumption decreased by 1.5 percentage points (2). All these factors affected the significant reduction in EI and CI.

The Ninth Five-Year Plan (1996–2000). The CI and EI further enhanced the negative effect compared to that in the Eighth FYP period. The PCG effect weakened carbon emission promotion, while the P effect maintained the same positive level as that before. Owing to the policy that was implemented to close down the 15 major categories of small heavy-pollution enterprises, the Southeast Asia financial crisis, and severe flood disasters, the demand for export products significantly declined. As such, economic growth was seriously affected by the weak export demand (4). The annual average GDP growth rate in the Ninth FYP was 8.4% (1). The Chinese government began to pay attention to environmental protection, encouraged the development of renewable energy, and issued the Energy Conservation Law (5), among others. Therefore, the proportion of coal consumption decreased by 5 percentage points (2) while energy efficiency was substantially increased and EI and CI further declined.

The Tenth Five-Year Plan (2001–2005). This was the only period where the effects of all four indicators were positive. Owing to the improvement of the international economic situation, the Chinese economy slowly improved. In 2001, China formally joined the WTO. As such, the Chinese economy entered a new era of investment and export-led economic growth. Consequently, the annual average growth of the GDP was 9.8% (1), which was higher than that of the Ninth FYP

period. To meet the needs of the international market, China invested heavily in energy-intensive industries, thereby leading to a rapid increase in the proportion of secondary industry, a sharp increase in energy consumption, and a significant rebound in EI (5). More seriously, the trend of energy structure optimization was reversed, the proportion of coal consumption increased by 4.4 percentage points (2), and the CI significantly increased.

The Eleventh Five-Year Plan (2006–2010). The CI and EI effects on carbon emissions became strongly negative compared to those during the Tenth FYP period. The PCG effect remained significantly positive, while the P effect remained at the same positive level as that before. Even though the global financial turmoil in 2008 had a serious impact on the global economy, China took the lead in the global economic uptrend after launching a policy package to expand domestic demand and promote economic growth; its annual average growth rate of GDP reached 11.2% (1), which was higher than the level in the Tenth FYP period. The excessive energy consumption exposed in the Tenth FYP period impelled the Chinese government to focus on energy conservation (5) and to list the energy consumption per unit of GDP as a binding target for national economic and social development planning. China’s Copenhagen pledge demonstrated its ambition to actively respond to climate change. Finally, the proportion of secondary industry decreased, the continued high carbonization trend of the energy structure was reversed, the proportion of coal consumption decreased by 3.2 percentage points (2), and the EI and CI significantly decreased.

The Twelfth Five-Year Plan (2011–2015). The negative effects of CI and EI during the Twelfth FYP were further enhanced compared to those during the Eleventh FYP. The PCG effect weakened carbon emission promotion, while the P effect remained at the same positive level as before. Since China’s economy entered a “new normal” stage with significant changes in key elements supporting rapid economic growth, economic growth faced pressures of transformation, upgrading, and restructuring. This led to the annual average growth rate of GDP declining to 7.8% (1), which was significantly lower than that of the Eleventh FYP period. However, the Chinese government achieved positive results in adjusting the industrial structure, optimizing the energy structure, and improving energy efficiency. The proportion of secondary industry decreased by 5.5 percentage points, non-fossil fuel energy consumption increased by 3.2 percentage points, coal consumption decreased by 6.5 percentage points (2), and the EI and CI were further

reduced.

The Thirteenth Five-Year Plan (2016–2018). The role of the CI and EI effects in carbon emission reduction was further enhanced compared with that in the same period of the Twelfth FYP (2011–2013) (Table S3). Meanwhile, the stimulating role of the PCG effect on carbon emissions decreased compared to that during the Twelfth FYP period, which was mainly because supply-side structural reform, the development of a philosophy of innovation and coordination, greening, opening-up, and sharing led to increased development of the Chinese economy (6). In the first 3 years of the Thirteenth FYP, the economic development growth rate remained at 6.7%, and in 2018, the proportion of secondary industry fell to 40.7%. Through the comprehensive promotion of energy production and consumption revolution, a new pattern of energy development using low-carbon energy to meet new energy demand was formed, and the energy structure was further optimized. In 2018, the proportion of non-fossil fuel energy consumption increased to 14.3%, and the proportion of coal consumption was further reduced to 59.0% (7). Table 1 provides an overview of the major trends outlined above.

Table S3 Determinant effects of four indicators from 1978 to 2018 (Mt CO_2)

Year	Net effect	Carbon intensity effect	Energy intensity effect	Per capita GDP effect	Population size effect
1979	34	-1	-67	83	18
1980	39	-1	-66	90	17
**1979-1980**	**73**	**-2**	**-133**	**173**	**35**
1981	-23	-4	-91	51	20
1982	62	-1	-62	102	23
1983	91	-3	-62	136	20
1984	126	10	-115	209	21
1985	141	3	-84	197	25
**1981-1985**	**396**	**5**	**-413**	**695**	**109**
1986	103	3	-61	131	29
1987	141	3	-83	189	33
1988	152	-1	-76	195	34
1989	94	0	1	60	34
1990	35	-8	-47	56	34
**1986-1990**	**525**	**-3**	**-267**	**631**	**164**
1991	128	6	-94	184	31
1992	125	-4	-210	309	30

1993	150	-13	-187	319	31
1994	150	-10	-186	315	32
1995	187	-13	-114	282	32
**1991-1995**	**739**	**-34**	**-790**	**1409**	**155**
1996	089	-5	-202	264	33
1997	-10	-27	-263	248	32
1998	0	-7	-232	210	29
1999	116	14	-137	214	26
2000	79	-60	-133	247	25
**1996-2000**	**274**	**-86**	**-968**	**1182**	**146**
2001	165	-40	-70	252	24
2002	332	12	-3	299	24
2003	679	48	231	375	25
2004	757	-9	292	444	29
2005	758	39	106	580	33
**2001-2005**	**2692**	**50**	**557**	**1949**	**135**
2006	579	-4	-177	726	34
2007	570	-9	-341	884	36
2008	129	-83	-463	637	37
2009	349	-6	-320	639	37
2010	437	-120	-240	759	38
**2006-2010**	**2063**	**-221**	**-1540**	**3644**	**181**
2011	687	89	-170	728	41
2012	193	-148	-337	633	44
2013	253	-77	-356	641	45
2014	50	-146	-458	606	48
2015	-32	-121	-531	574	46
**2011-2015**	**1150**	**-403**	**-1853**	**3183**	**224**
2016	-29	-155	-474	546	54
2017	177	-102	-345	573	50
2018	214	-100	-296	573	36
**2016-2018**	**362**	**-356**	**-1115**	**1693**	**140**
**1978-2018**	**8275**	**-1050**	**-6523**	**14559**	**1289**

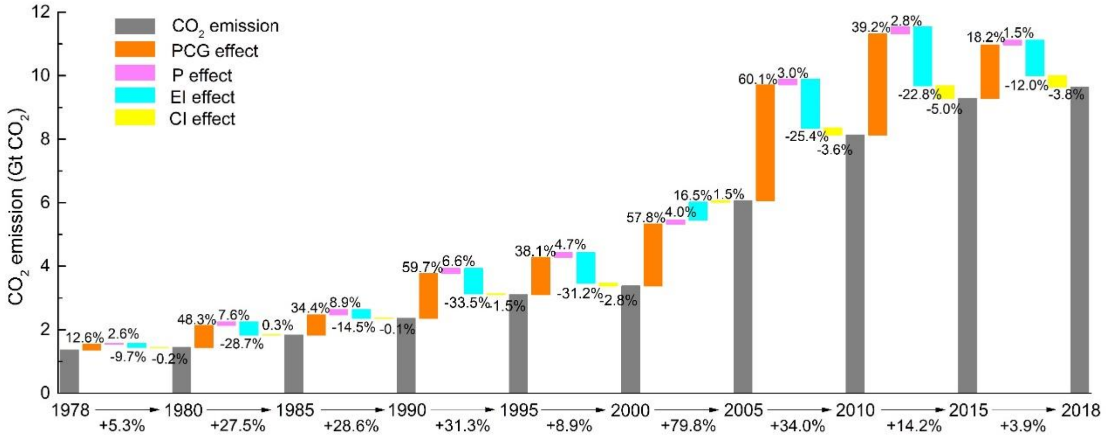

Figure S2. Determinant effects of four indicators from 1978 to 2018 in terms of the five-year plans. The grey column represents China's CO_2 emissions over 5-year time span, and the coloured columns represent the contribution to the change in CO_2 emissions during each five-year plan period. The percentage numbers above the *x* axis refer to the determinant's contributions to the growth rate changes in CO_2 emissions over 5-year time span, and the contributions can be positive or negative. The percentage numbers below the *x* axis refer to CO_2 emission changes over 5-year time span.

Table S4 Results of Augmented Dickey-Fuller (ADF) test for five variables

	Variables	Method (c, t, p)	ADF value	p value	Conclusion
Level	lnCE	(1, 1, 1)	-3.104174	0.1195	Non-stationary
	lnCI	(0, 0, 0)	-2.859071	0.0054*	Stationary
	lnEI	(0, 0, 0)	-3.829601	0.0003*	Stationary
	lnPCG	(1, 1, 2)	-3.847312	0.0243*	Stationary
	lnPR	(0, 0, 2)	2.557793	0.9968	Non-stationary
First difference	lnCE	(1, 0, 1)	-2.393327	0.1501	Non-stationary
	lnCI	(1, 1, 0)	-6.162245	0.0000*	Stationary
	lnEI	(1,0, 0)	-2.379869	0.1538	Non-stationary
	lnPCG	(1, 0, 2)	-4.080965	0.0029*	Stationary
	lnPR	(1, 0, 2)	-5.579129	0.0000*	Stationary
Second difference	lnCE	(0, 0, 1)	-6.069838	0.0000*	Stationary
	lnCI	(0, 0, 0)	-12.43654	0.0000*	Stationary
	lnEI	(0, 0, 0)	-4.859631	0.0000*	Stationary
	lnPCG	(0, 0, 2)	-6.151807	0.0000*	Stationary
	lnPR	(0, 0, 2)	-7.119675	0.0000*	Stationary

Note: c represents intercept, there is an intercept, if c value is 1; no intercept, if c value is 0. t represents trend, there is trend, if t value is 1; no trend, if t value is 0. p represents lags.

Table S5 Information criteria for selecting optimal lag of unrestricted VAR

Lag	LogL	LR	FPE	AIC	SC	HQ
1	606.9977	NA	7.56e-20	-29.84604	-28.77965	-29.46343
2	672.5255	97.45161*	9.96e-21*	-31.92439*	-29.79161*	-31.15917*

* indicates lag order selected by the criterion

LR: sequential modified LR test statistics (at 5% level)

FPE: Final prediction error

AIC: Akaike information criterion

SC: Schwarz information criterion

HQ: Hannan-Quinn information criterion

Table S6 Unrestricted Cointegration Rank Test (Maximum Eigenvalue)

Hypothesized No. of CE(s)	Eigenvalue	Max-Eigen Statistic	0.05 Critical Value	Prob.**
None *	0.584584	34.26053	33.87687	0.0450
At most 1	0.412010	20.71074	27.58434	0.2941
At most 2	0.366661	17.81322	21.13162	0.1370
At most 3	0.248982	11.16669	14.26460	0.1461
At most 4	0.004748	0.185613	3.841466	0.6666

CE stands for cointegration equation; Max-eigenvalue test indicates 1 cointegrating equations at the 0.05 level

* denotes rejection of the hypothesis at the 0.05 level

**MacKinnon-Haug-Michelis (1999) p-values

References

1. National Bureau of Statistics of China. *China Statistical Yearbook 2018*. *China Statistics Press*, Beijing, (2018).
2. National Bureau of Statistics of China. *China Energy Statistical Yearbook 2018*. *China Statistics Press*, Beijing, (2018).
3. Wu, J. & Zhang, Y. L. Looking back 30 years: research on system of household contract responsibility. *Economic Theory and Business Management* **27**, 43-47 (2008).
4. Guan, H. H. & Lin, Z. X. Five-year plans and history of China's economy. Available at http://news.hexun.com/2011-04-20/128901983_2.html (2011).
5. Yu, C. et al. Great achievements of energy conservation since 1978 reform and opening up. *Energy of China* **40**, 4–8 (2018).
6. Hu, A. G. et al. The strategic innovations towards adapting to and leading China’s new normal status. *Journal of Tsinghua University* **31**, 17-22 (2016).
7. National Bureau of Statistics of China. China Statistical Abstract 2019. *China Statistics Press*, Beijing, (2019).
